# Comparison of Pediatric Sequential Organ Failure Assessment and Pediatric Risk of Mortality III Score as Mortality Prediction in Pediatric Intensive Care Unit

**DOI:** 10.7759/cureus.21055

**Published:** 2022-01-09

**Authors:** Sadam H Baloch, Ikramullah Shaikh, Murtaza A Gowa, Pooja D Lohano, Mohsina N Ibrahim

**Affiliations:** 1 Pediatric Medicine, National Institute of Child Health, Karachi, PAK; 2 Pediatric Critical Care, National Institute of Child Health, Karachi, PAK; 3 Pediatrics and Endocrinology, National Institute of Child Health, Karachi, PAK

**Keywords:** pediatric risk of mortality (prism) iii score, pediatric sequential organ failure assessment (p-sofa), pediatric intensive care unit, mortality, children

## Abstract

Objective: To assess and compare the diagnostic accuracy of the Pediatric Risk of Mortality (PRISM) III score and Pediatric Sequential Organ Failure Assessment (p-SOFA) for the prediction of mortality in critically ill children.

Methodology: This was a cross-validation study conducted at the Pediatric Intensive Care Unit (PICU) of the National Institute of Child Health Karachi from February 2021 to July 2021. Two hundred eighty-six critically ill children of age one month to 15 years of either gender staying in PICU for more than 24 hours were included. Within 24 hours of admission, the p-SOFA and PRISM III 24 scores were calculated for all eligible children. The outcome of the study was mortality within 30 days of PICU admitted children. Data were analyzed using Statistical Package for the Social Sciences (SPSS) version 23.

Results: The median age was 24 months (range: 1-144 months). The 30-day mortality was estimated as 57%. The p-SOFA and PRISM scores were significantly greater in children who did not survive than survivors. The maximum p-SOFA score (area under the curve (AUC)=0.81, 95% CI=0.76-0.86, p=0.001) and PRISM III 24 score (AUC=0.75, 95% CI=0.69-0.81, p=0.001) had good discrimination for 30-day mortality. For the prediction of 30-day mortality at the cut-off value of p-SOFA>2, the sensitivity was 93.87%, specificity was 38.21%, and accuracy was 69.93%. Whereas at the cut-off value of PRISM III 24 score>8, the sensitivity was 55.83%, specificity was 77.24%, and accuracy was 65.03%.

Conclusion: The p-SOFA score is a good predictor for 30-day mortality in critically ill children and had better accuracy than the PRISM III 24 score.

## Introduction

The pediatric intensive care unit (PICU) plays an important role in delivering demanding and required care to seriously ill children. In both developing and developed countries, PICU children have a considerably higher risk of morbidity and death [1,2]. Quality and quantities of PICUs are improving in developing countries like Pakistan, but it is an uphill process, as the units need modern, expensive equipment and a large highly trained staff. So there is a need for time to employ methods, techniques, and scoring systems that are predictive of mortality and morbidity risk in these patients, thus allowing these systems to assist in timely and focused decisions regarding the deployment of different expertise and resources, to produce highly productive results [3,4].

Several prognostic scoring systems like Pediatric Index of Mortality (PIM and PIM2), Pediatric Risk of Mortality (PRISM, PRISM III), Sequential Organ Failure Assessment (SOFA), Pediatric Sequential Organ Failure Assessment Score (p-SOFA), and the Paediatric Logistic Organ Dysfunction (PELOD) score have been developed to predict PICU children's morbidity and death, which can be extremely helpful in treatment planning [1,2,5-9]. The PRISM III 24 score is a commonly used system that is used to evaluate various scoring systems. PRISM III 24 score helps us in predicting institutional performance [10,11]. Models like the PRISM III 24 score provide one of the best ways to organize an intensive care unit. PRISM III 24 score takes 24 hours to complete and cannot be used in regulating admissions to the PICU but only to assess illness severity and length of stay [3,12]. The p-SOFA has been developed recently and so far only validated retrospectively in critically ill children [13,14]. Whereas only a few studies have been conducted for validation of p-SOFA and PRISM III in developing countries like Pakistan. An ideal scoring system would be accurate, easy to use, and simple, as well as minimally intrusive and low-cost. However, no scoring system is flawless, and each one has its own set of limitations, which is why studies are being conducted to enhance accuracy, verify existing ones, and develop new ones [15,16]. Therefore, the aim of our study was to assess and compare the diagnostic accuracy of the PRISM III and the p-SOFA for the prediction of mortality in critically ill children.

## Materials and methods

From February to July 2021, cross-validation research was done in the PICU of the National Institute of Child Health (NICH) in Karachi. This study was carried out with the approval of NICH Karachi's Institutional Ethical Review Board, approval number 42/2020, and with the signed informed consent of the children's parents. The sample size was calculated using the diagnostic accuracy sample size calculator by Dr. Lin Naing, with PRISM sensitivity of 70.6%, specificity of 82.3%, a margin of error of 5.2%, 30-day mortality prevalence of 28.1%, and a confidence level of 95%. The sample size was calculated to be 286 [2]. A non-probability consecutive sampling approach was used to include critically ill children aged one month to 15 years of either gender who had been in the PICU for more than 24 hours. Patients with an underlying congenital deformity who were hospitalized for routine treatment before planned procedures such as intravenous immunoglobulin (IVIG) and hemodialysis, cardiopulmonary resuscitation (CPR) before admission, and children who died within 12 hours of admission were excluded from the research. Within 24 hours of admission, the p-SOFA score was calculated for all eligible children. p-SOFA score of higher than 2 is considered as a predictor of mortality. We calculated PRISM III score within 24 hours after admission, in addition to p-SOFA. A score of higher than 8 on the PRISM III 24 was deemed a predictor of mortality. On a pre-designed proforma, demographic data such as age, weight, type of admission (clinical or medical cases and surgical (post-surgical recovery cases)), length of stay in PICU, need for ventilator support, and inotropic support was also noted by the researcher himself. All of the children were followed up from admission till discharged from the PICU. Mortality within 30 days was the outcome of the study.

After collecting data the analyses were conducted by using Statistical Package for Social Sciences (SPSS) version 23 (IBM Corp., Armonk, NY, USA). We presented our numeric data with median and range as they were non-normally distributed. We presented our categorical variables with frequencies and percentages. We compared numeric data with the Mann-Whitney U test and categorical data with the chi-square test to assess the association with mortality. PRISM III 24 and p-SOFA scores were categorized according to the 25th, 50th, and 75th percentiles of these scores. Comparison between categories of PRISM III 24 score and p-SOFA with 30-day mortality were done using Pearson’s chi-square test. Two by two tables were used to calculate the specificity (Sp), sensitivity (Sn), negative predictive value (NPV), positive predictive value (PPV), and accuracy of PRISM III 24 and p-SOFA scores by taking 30-day mortality as the gold standard. Spearmen’s correlation was applied to assess the relationship between the duration of PICU stay and both scores (PRISM III 24 score and p-SOFA score). A p-value of ≤0.05 was taken as statistically significant.

## Results

We included 286 children in the study. The median age was 24 months (range: 1-144 months). The 30-day mortality was estimated as 57%. The median age of 163 non-survivors was 24 months (range: 2-144 months). Table 1 displays the other information of non-survivors and survivors.

**Table 1:** Baseline information of study sample (n=286) PRISM: Pediatric Risk of Mortality; p-SOFA: Pediatric Sequential Organ Failure Assessment; PICU: Pediatric Intensive Care Unit

	Mortality		Total	p-value	
	Yes (n=163)	No (n=123)			
Age (months)	24 (2-144)	21 (1-144)	24 (1-144)	0.902	
Weight (kg)	10 (2.2-40)	10 (2.7-47)	10 (2.2-47)	0.578	
Type of admission					
Clinical	155 (95.1)	108 (87.8)	263 (92)	0.025*	
Surgical	8 (4.9)	15 (12.2)	23 (8)		
Ventilator requirement					
Yes	163 (100)	79 (64.2)	242 (84.6)	0.001*	
No	0	44 (35.8)	44 (15.4)		
Inotrope Need					
Yes	140 (85.9)	27 (22)	167 (58.4)	0.001*	
No	23 (14.1)	96 (78)	119 (41.6)		
p-SOFA score	8 (1-20)	4 (0-14)	6.50 (0-20)	0.001*	
PRISM III 24 score	10 (0-29)	3 (0-20)	7 (0-29)	0.001*	
Duration of PICU stay (days)	5 (1-40)	5 (1-39)	5 (1-40)	0.679	
Data expressed as median (range: minimum-maximum), or n (%). *Statistically significant					

**Figure 1:**
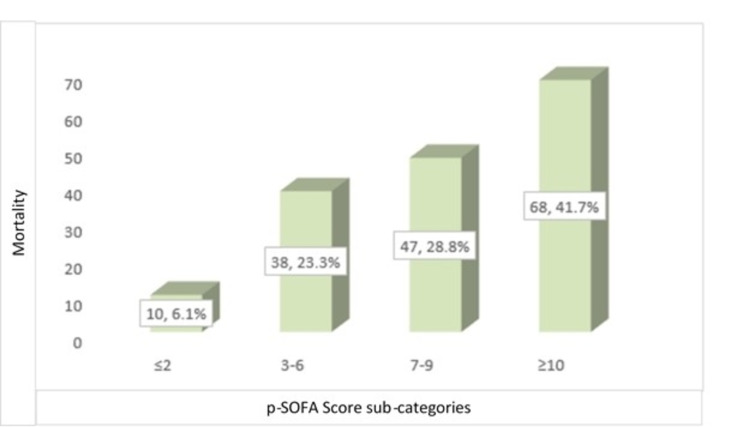
Frequency distribution of 30-day mortality among the different categories of p-SOFA: The x-axis displays p-SOFA sub-categories. Children were categorized based on cut off values corresponding to the 25th, 50th and 75th percentiles in the distribution of p-SOFA scores. The y-axis displays frequency of children that died in subcategory relative to total number of mortalities. p-SOFA: Pediatric Sequential Organ Failure Assessment

We found that children who did not survive had a significantly higher p-SOFA score than survivors (8 [range: 1-20] vs 4 [range: 0-14]; p=0.0001; Table 1). We next divided the children into three groups based on the p-SOFA score's 25th, 50th, and 75th percentiles, and found that the frequency of death increased substantially with the p-SOFA score (p=0.001) (Figure 1).

**Figure 2:**
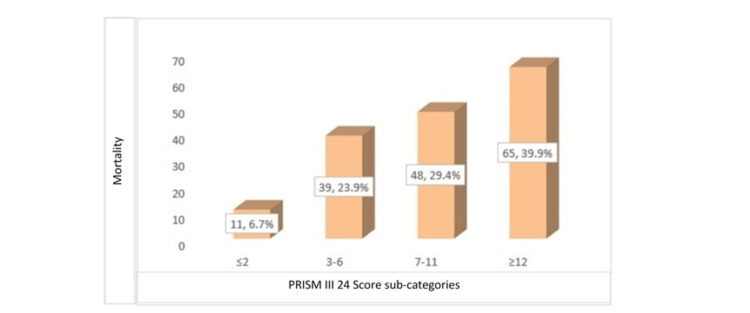
Frequency of distribution of 30-day mortality among different categories of PRISM III 24: The x-axis displays PRSIM III 24 score subcategories. Children were categorized based on cut off values corresponding to the 25th, 50th and 75th percentiles in the distribution of PRISM III 24 scores. The y-axis displays the frequency of children that died in sub-categories relative to total number of mortalities. PRISM: Pediatric Risk of Mortality

We found that PRISM III 24 score was significantly greater in children who did not survive than survivors (10 [range: 0-29] vs 3 [range: 0-20]; p=0.0001; Table 1). We further grouped children based on the 25th, 50th, and 75th percentiles of the PRISM III 24 score, we observed that frequency of mortality increased significantly with the PRISM III 24 score (p=0.001) (Figure 2).

**Figure 3:**
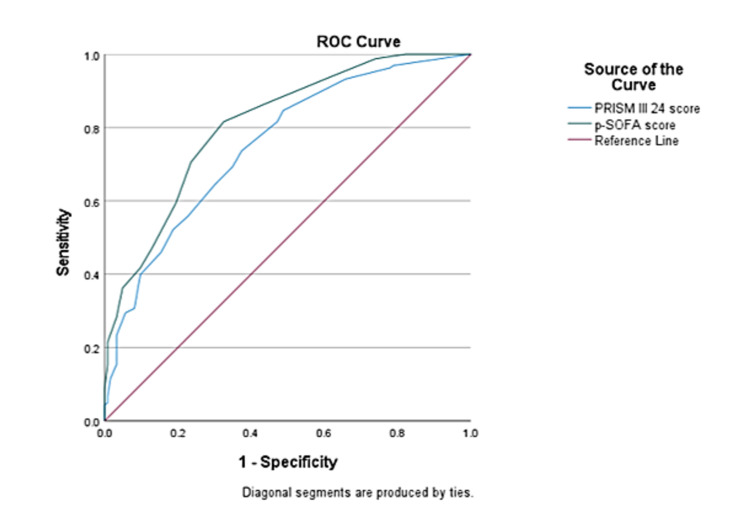
ROC curves analysis for relation of 30-day mortality using p-SOFA and PRISM III 24 scores in critically ill patients. PRISM: Pediatric Risk of Mortality; p-SOFA: Pediatric Sequential Organ Failure Assessment; ROC: receiver operating characteristic

With an area under the curve (AUC) of 0.81 (0.76-0.86) and a p-value of 0.0001, the maximal p-SOFA exhibited good discrimination for 30-day mortality. The maximum PRISM III 24 had good discrimination for 30-day mortality, with the AUC of 0.75 (0.69-0.81) with a p-value=0.0001 (Figure 3).

For the prediction of 30-day mortality at the cut-off value of p-SOFA>2, the sensitivity was 93.87%, specificity was 38.21%, PPV was 66.81%, NPV was 82.46% and accuracy was 69.93%. Whereas at the cut-off value of PRISM III 24 score>8, the sensitivity was 55.83%, specificity was 77.24%, PPV was 76.47%, NPV was 56.89% and accuracy was 65.03% (Table 2).

**Table 2:** Correlation analysis between PRISM III 24 score and p-SOFA score and duration of PICU stay (n=286) PRISM: Pediatric Risk of Mortality; p-SOFA: Pediatric Sequential Organ Failure Assessment; PICU: Pediatric Intensive Care Unit

	PRISM III 24 score	p-SOFA score	Duration of PICU stay
PRISM III 24 score	1	0.712 (0.001*)	0.063 (0.290)
p-SOFA score	0.712 (0.001*)	1	0.054 (0.366)
Duration of PICU stay	0.063 (0.290)	0.054 (0.366)	1
Data expressed as Spearmen's rho (p-value)			
*Statistically significant			

We observed that the p-SOFA score was positively associated with the PRISM III 24 score (r=0.712, p=0.001). No statistically significant correlation was found between PRISM III 24 score and duration of stay as well as between p-SOFA score and duration of hospital stay (Table 3).

**Table 3:** Diagnostic accuracy of p-SOFA and PRISM III 24 score by taking 30-day mortality (n=286) PRISM: Pediatric Risk of Mortality; p-SOFA: Pediatric Sequential Organ Failure Assessment; PPV: positive predictive value; NPV: negative predictive value

	30-day mortality		Statistics
	Yes	No	
p-SOFA score			Chi-square test=45.19 (p=0.001), Sensitivity=93.87%, Specificity=38.21%, PPV=66.81%, NPV=82.46%, Accuracy=69.93%
> 2	153 (93.9%)	76 (61.8%)	
≤ 2	10 (6.1%)	47 (38.2%)	
PRISM III 24 score			
> 8	91 (55.8%)	28 (22.8%)	Chi-square test=31.54 (p=0.001), Sensitivity=55.83%, Specificity=77.24%, PPV=76.47%, NPV=56.89%, Accuracy=65.03%
≤ 8	72 (44.2%)	95 (77.2%)	

## Discussion

In the PICU, scoring systems help healthcare managers determine a patient's prognosis and improve the decision-making process as well as the patient's outcome [9,17]. In a country like Pakistan, where tertiary care institutions are few, the outcome of PICU estimates the most effective use of limited resources and helps guide policy formation [3]. The use of scoring systems to predict PICU outcomes can also aid in prompt intervention [18]. However, developing the optimal scoring method for critically ill children admitted to the PICU remains a challenging task [19]. Different methods are used in PICUs for the prediction of severity of disease and risk of mortality, including Pediatric Index of Mortality (PIM and PIM2), Pediatric Risk of Mortality (PRISM, PRISM III), Sequential Organ Failure Assessment (SOFA), Pediatric Sequential Organ Failure Assessment Score (p-SOFA), and the Paediatric Logistic Organ Dysfunction (PELOD) score [1,2,5-9]. Although these scoring systems are effective in ICUs all over the world, it is important to evaluate them before using them in healthcare settings that are very different from where they were established [3,20,21]. Until now, the validation of the p-SOFA and PRISM III 24 scoring systems in Pakistani PICUs has not been extensively examined. Therefore, in this study, we evaluated the validity of the PRISM III 24 score and the p-SOFA score for predicting 30-day death in 286 critically ill children admitted to the PICU at a tertiary care hospital in Karachi. The 30-day death rate was predicted to be 57% in the current study, with the median age of non-survivors being 24 months. The higher number of clinical cases (92%) admitted to PICU might be the reason for the higher mortality rate. Similarly, in a Pakistani study, Quershi et al. [4] discovered that the median age of PICU children was 18 months and that the death rate was 28.7%, which was also higher than mortality rates documented by other validation studies. In addition, they also had a larger percentage of non-surgical patients (86%) in their research. Similarly, in an Indian study of 75 PICU children, Kumbar et al. discovered that 37.3% of the children died. They also discovered that older children (aged >15 years) had a greater death rate, whereas younger children had a higher survival rate. They did not, however, find a statistically significant association between age and death (p=0.513) [18].

In the current study, p-SOFA and PRISM III 24 scores at the time of admission to the PICU were found to be good predictors of death within 30 days. The probability of mortality increased in a stepwise way from smaller to larger p-SOFA and PRISM III 24 scores across patient sub-categories, with survivors having a significantly lower median p-SOFA and PRISM III 24 scores than non-survivors. Furthermore, the AUC value of 0.81 for the prediction of 30-day mortality was good for the p-SOFA score. This finding is comparable to that of El-Mashad et al., who reported a good AUC of 0.89 for the prediction of 30-day death in PICU children using the p-SOFA score [2]. Similarly, Matics et al. discovered a good AUC of 0.88 for the prediction of 30-day death in PICU infants using SOFA score in their investigation [14]. The PRISM III 24 score, on the other hand, had a lower AUC value of 0.75 for predicting 30-day mortality than the p-SOFA AUC value in the current investigation, according to ROC curve analysis [14]. Similarly, El-Mashad et al. also found that the SOFA score had a higher AUC than the PRISM score for the prediction of 30-day mortality (0.89 vs 0.84) in PICU children [2]. Another study by Zhou et al. also found that p-SOFA had higher predictive accuracy for the prognosis of PICU children with severe illness than PRISM III [22]. Lalitha et al. also found that p-SOFA had good accuracy (AUC=0.84) as compared to PRISM III (AUC=0.70) for the prediction of mortality [23]. In this study, we used >8 and >2 as the optimum PRISM III 24 and p-SOFA cut-off values for the prediction of 30-day mortality. We found that p-SOFA (>2) had a greater sensitivity than PRISM III 24 (>8) (94% vs. 56%), while p-SOFA (>2) had a lower specificity than PRISM III 24 (>8) (38.2% vs 77.2%). Furthermore, p-SOFA (>2) had somewhat better accuracy than PRISM III 24 (>8) (70% vs 65%). El-Mashad et al. also discovered that the SOFA is more accurate than the PRISM score in predicting death in PICU children. They discovered that a SOFA score of more than 2 predicted death in 40% of PICU patients with 100% sensitivity and 5.2% specificity. At higher cut-off values of scoring systems for the prediction of 30-day mortality, they found p-SOFA score (cut-off >6.5) had sensitivity as 80.9% and specificity as 81.8%, whereas PRISM score (cut-off >17.2) had sensitivity as 70.6% and specificity as 82.3% [2]. We also found a positive correlation between the p-SOFA and PRISM III 24 score in PICU admitted children (r=0.712). El-Mashad et al. also revealed that the p-SOFA score was positively correlated with the PRISM score (r=0.59) [2].

Apart from the fact that p-SOFA was a better predictor of 30-day death among critically ill children than PRISM III in our study, the p-SOFA score offers other advantages over PRISM III. p-SOFA is free of cost and does not require the use of a calculator. p-SOFA has fewer calculation parameters than the PRISM III 24 score. p-SOFA is computed every day, providing a dynamic assessment of the progression of the disease, whereas PRISM is performed only at the time of admission. Because it is tailored to oxygen saturation (SPO2) values rather than partial pressure of oxygen (PaO2) readings, the p-SOFA does not need arterial blood gas analysis, which can be difficult to acquire in children [6,23-25]. With limited resources, there are just a few tertiary care referral institutions for pediatric admission in Pakistan. In such circumstances, p-SOFA can be used to determine which patients require critical care and can also aid in predicting the patients' prognosis. There are several limitations to our research study. All the data was collected from a single PICU, in comparison to other validation studies, the present study's sample size was relatively small. More studies with a larger sample size are required in the future to improve the accuracy of results. 

## Conclusions

Mortality among the children admitted to critical care units is high despite the use of advanced equipment and sophisticated techniques. p-SOFA and PRISM III 24 both have proven to be valuable in objective prediction in these patients. In this study, we compared the diagnostic accuracy of the Pediatric Risk of Mortality (PRISM) III score and Pediatric Sequential Organ Failure Assessment (p-SOFA) for the prediction of mortality in critically ill children and showed that the p-SOFA score is a good predictor for 30-day mortality in critically ill children with better accuracy than the PRISM III 24 score.
